# Is CMV a target in pediatric glioblastoma? Expression of CMV proteins, pp65 and IE1-72 and CMV nucleic acids in a cohort of pediatric glioblastoma patients

**DOI:** 10.1007/s11060-015-1905-z

**Published:** 2015-09-04

**Authors:** Amanda Wakefield, Antonella Pignata, Alexia Ghazi, Aidin Ashoori, Meenakshi Hegde, Daniel Landi, Tara Gray, Michael E. Scheurer, Murali Chintagumpala, Adekunle Adesina, Stephen Gottschalk, John Hicks, Suzanne Z. Powell, Nabil Ahmed

**Affiliations:** Texas Children’s Hospital, Houston Methodist Hospital, Center for Cell and Gene Therapy, Baylor College of Medicine, 1102 Bates Street MC 3-3320, Houston, TX 77030 USA; Texas Children’s Hospital, Texas Children’s Cancer and Hematology Centers, Baylor College of Medicine, Houston, TX 77030 USA; Departments of Pediatrics, Baylor College of Medicine, Houston, TX 77030 USA; Departments of Pathology and Immunology, Baylor College of Medicine, Houston, TX 77030 USA; Department of Pathology and Genomic Medicine, Houston Methodist Hospital, Houston, TX 77030 USA

**Keywords:** Glioblastoma, GBM, Pediatric, CMV, pp65, IE1-72

## Abstract

While the 5-year overall survival is better in pediatric than in adult patients diagnosed with glioblastoma (GBM), outcomes in children remain very poor. Understanding the mechanisms of tumorigenesis and tumor propagation can identify therapeutic targets to improve these outcomes. Human cytomegalovirus (CMV) proteins and nucleic acids are present in the majority of adult GBM. Indeed, CMV is emerging as a potential glioma-associated target for anti-CMV agents and cellular therapeutics. Furthermore, CMV appears to contribute to GBM’s malignant phenotype, although its role in tumorigenesis is less certain. In this cohort of 25 serially diagnosed pediatric GBMs, the largest described cohort to date, we used immunohistochemical staining and in situ hybridization to show the presence of CMV antigens pp65 and IE1-72 as well as CMV nucleic acids, respectively. Our cohort indicated either CMV antigen pp65 or IE1-72 was present in approximately 67 % of pediatric GBM samples. The majority of samples stained positive for either CMV antigen showing a cytoplasmic pattern in 25-50 % of cells within the sample at a moderate intensity, while a few samples showed nuclear staining and higher grade/intensity. Of 16 samples where in situ hybridization was performed, 13 (81 %) showed specific staining using a CMV genome specific probe cocktail. ISH positive samples showed high concordance with being pp65 or IE1-72 positive. These findings, paired with the association of CMV expression with poor prognosis and overall survival, indicate the need to further investigate how these antigens are promoting tumor growth and preventing cell death. Also, the expression of these antigens in a majority of tumor tissues should be considered for immunotherapeutic targets in cases of pediatric GBM.

## Introduction

In children, approximately 65 % of glioblastoma (GBM) arise in the cerebrum, 20 % in the thalamus and hypothalamus, and 15 % in the posterior fossa, mostly affecting the cerebellum and brainstem [1]. While GBM in both pediatric and adult patients represents the most anaplastic and highest grade of gliomas, these tumors appear to differ in their genetic and molecular underpinnings [1]. Current treatment includes tumor resection, radiotherapy, and occasionally in children, adjuvant chemotherapy. This combination is both toxic and largely ineffective [2, 3].

GBMs exhibit numerous sophisticated defense mechanisms making them resistant to conventional therapies. They are notorious for microscopically infiltrating healthy brain, making complete resection difficult. In addition, glioma stem cells (GSC) are quiescent and appear to have advanced DNA repair mechanisms, anti-apoptosis genes, and telomerase activity, rendering them resistant to both chemotherapy and radiation [3].

More effective therapies for GBM are needed, thus cellular therapies are being developed. Cytotoxic T lymphocytes (CTLs) are powerful immune effector cells and have been successfully used to treat disseminated Epstein–Barr Virus (EBV) infections and EBV-driven malignancies [4, 5]. CTLs can be directed to target GBM through ex vivo expansion of a tumor-specific clone or manipulation of their T cell receptor (TCR), but effective immunogenic targets are strongly needed [6–8]. Through identifying unique targets for cellular therapies and increasing understanding of tumor escape mechanisms, we and others are developing more specific and powerful therapies for GBM that effectively target tumor cells while sparing the intricate neighboring healthy tissue [6–8].

Recently, the detection of CMV proteins and nucleic acid in the majority of adult GBM has caused interest in these as a possible target for immune-based biologics [9–14]. Initial techniques for detecting CMV proteins and nucleic acids were varied, but under optimal conditions CMV proteins are found in the majority of high-grade gliomas [14]. CMV early or late proteins have also been found in up to 100 % of neuroblastomas [15] and 40 % of medulloblastomas [16]. CMV has been implicated in promoting GBM pathogenesis; specifically, CMV appears to enhance telomerase activity and angiogenesis in adult GBM [17–19]. Increased awareness of the prevalence of CMV expression on GBM tumor cells and its apparent ability to enhance tumor cell survival and invasiveness make it an appealing target for immunotherapy against GBM.

Several reports describe the prevalence of CMV expression patterns in adult cancers, including adult GBM, and support the potential for CMV as an immunotherapeutic target [9–14]. By determining the expression pattern of CMV antigens in pediatric GBM, we can discern their potential as a target for cellular and other targeted therapies, as well as pursue a better understanding of their roles in tumor pathogenesis. Ultimately, this knowledge could allow us to better determine the utility of CMV for improving survival in children with GBM.

## Materials and methods

### Study subjects

GBM tissue samples from 15 pediatric patients were obtained from Texas Children’s Hospital; one of these samples was immeasurable due to necrotic tissue. The remaining 10 samples were sent from collaborating institutions for analysis. All tissue sections were from children under the age of 18 at the time of resection, and serially diagnosed GBM WHO grade IV. All patients were consented on a human protocol approved by Baylor College of Medicine’s internal review board (IRB). Seropositivity for CMV was unknown. Fifteen of 25 tumors were primary excisions and 10 were tumor recurrences.

### Standard staining

All tissues were received from collaborating institutions as recently cut 5 µm slides from tumor material fixed in 10 % formalin and paraffin embedded. H&E stains were also received completed by collaborating institutions.

### Immunohistochemistry (IHC)

To test samples for CMV, IHC staining was performed as previously described [20]. Briefly, Formalin-fixed, paraffin embedded sections (6 µm) of primary human GBM were used. (1) Known CMV-infected lung samples were used as a positive tissue control for all experiments. All slides were deparaffinized by heating slides in a xylene bath at 50 °C for 1 h and 10 min, followed by a 30-min incubation period at room temperature. The slides were then washed additionally in xylene and serially diluted in ethanol baths (100, 95, 70 and 50 %), post-fixed in neutral buffered formalin and treated for pepsin digestion (BioGenex, San Ramon, CA, USA). Freshly prepared 30 % H_2_O_2_ was used to block endogenous peroxidase before performing antigen retrieval using CitraPlus antigen retrieval solution (BioGenex) for 2.5 h at 50 °C. Avidin, biotin (BioGenex) and Fc receptor (Innovex Biosciences, Richmond, CA, USA) blocking reagents were applied to the sections prior to a 4 °C overnight incubation with anti-IE172 (1:100; Chemicon, Temecula, CA, USA) and anti-pp65 (1:40; Leica Microsystems Inc., Bannockburn, IL, USA) primary antibodies. Positive control sections were treated with anti-actin monoclonal antibody (1:35; BioGenex), while negative control sections were similarly incubated with no primary antibody. The sections were then developed using a biotinylated anti-mouse secondary antibody (1:16.5; BioGenex), peroxidase-labeled streptavidin (Biogenex) and 3,3′diaminobenzidine (Innovex Biosciences) as a chromogen. All slides were counterstained in Harris hematoxylin, dehydrated and coverslipped.

### In situ hybridization (ISH)

All paraffin-embedded sections (5 μm) were deparaffinized and post-fixed in neutral buffered formalin, similar to the sections for IHC. Pepsin digestion, endogenous peroxidase block and antigen retrieval were also performed for the ISH sections as previously described [20]. For the tests, CMV-infected lung tissue was used as a positive control. A human CMV DNA probe cocktail end-labeled with five fluorescein-linker molecules (Leica) was used to detect human CMV DNA in the sections. A probe against the reverse complementary sequence of black beetle virus RNA2 sequence (Leica) was used as a nonspecific negative control.

## Results

### Patient characteristics

A cohort of 25 pediatric patients are reported herein. These children, aged 9 months to 18 years (Table 1), were serially-diagnosed with GBM (WHO grade IV), and their tumors were procured at Texas Children’s Hospital and other institutions. Pathological diagnosis was confirmed by 3 independent pathologists (two neuropathologist and one pediatric neuropathologist). All patients were consented on a human protocol approved by Baylor College of Medicine’s institutional review board (IRB). Seropositivity for CMV was unknown. Fifteen of 25 tumors were primary excisions and 10 were tumor recurrences. Sixteen of 25 patients had enough material for both immunohistochemistry (IHC) and in situ hybridization (ISH).

**Table 1:** Patient and tumor characteristics

UPN	GBM grade	Location	Age	Gender
1	IV	Posterior fossa	11	M
2	IV	Frontal brain	4	F
3	IV	Frontal cortex	11	F
4	IV	Frontal brain	18	M
5	IV	Intraventricular	1	F
6	IV	Frontal brain	9	M
7	IV	Frontal cortex	11	M
8	IV	Thalamus	9	M
9	IV	Frontal brain	6	F
10	IV	Side brain	12	M
11	IV	Frontal brain	15	M
12	IV	Frontal brain	9 m	F
13	IV	Posterior fossa	10	M
14	IV	Posterior fossa	10	M
15	IV	Frontal brain	13	F
16	IV	Thalamus	17	M
17	IV	Left ventricle	13	M
18	IV	Frontal brain	4	F
19	IV	Front parietal	11	M
20	IV	Frontal brain	15	F
21	IV	Left anterior	15	M
22	IV	Frontal brain	18	M
23	IV	Thalamus	17	F
24	IV	Frontal brain	14	F
25	IV	Temporal	10	F

### Establishment of a grade and intensity scale for IHC

**Fig. 1:**
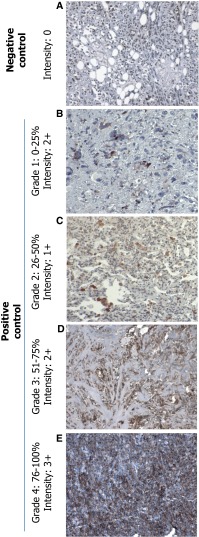
IHC staining showing representative grade and intensity scoring. **a** Sample with 0 % positive staining. **b** Sample staining positive at Grade: 1 (0–25 %) with Intensity: 2+. **c** Sample staining positive at Grade: 2 (26–50 %) with Intensity: 1+. **d** Sample staining positive at Grade: 3 (51–75 %) with Intensity: 2+. **e** Sample staining positive at Grade: 4 (76–100 %) with Intensity: 3+. Grade and intensity were measured by three independent pathologist for all tested patients. Magnification ×100

All IHC stains were given a grade (based on percent positivity) and an additional score for intensity of the stain using a pre-determined scheme (Fig. 1). All slides were reviewed for by 3 independent pathologists and the grading system was established by all pathologists and verified by independent sample review. Staining grade ranged from 1 to 4 with grade 1 indicating a positive stain visible in 1–25 % of cells, grade 2 in 26–50 % of cells, grade 3 in 51–75 % of cells, and grade 4 in 76–100 % of cells. Intensity ranged from 1+ to 3+ based on the positivity observed on control slides. Representatives for each grade and intensity are shown (Fig. 1) including a negative control (Fig. 1a). Grade distribution is shown increasing from grade 1 (Fig. 1b), grade 2 (Fig. 1c), grade 3 (Fig. 1d), and grade 4 (Fig. 1e). Intensity increases from 1+ (Fig. 1c), 2+ (Fig. 1d), and 3+ (Fig. 1e). There was no correlation between that staining grade or intensity and any particular pathological features.

### Expression of human cytomegalovirus (CMV) proteins, pp65 and IE1-72

**Fig. 2:**
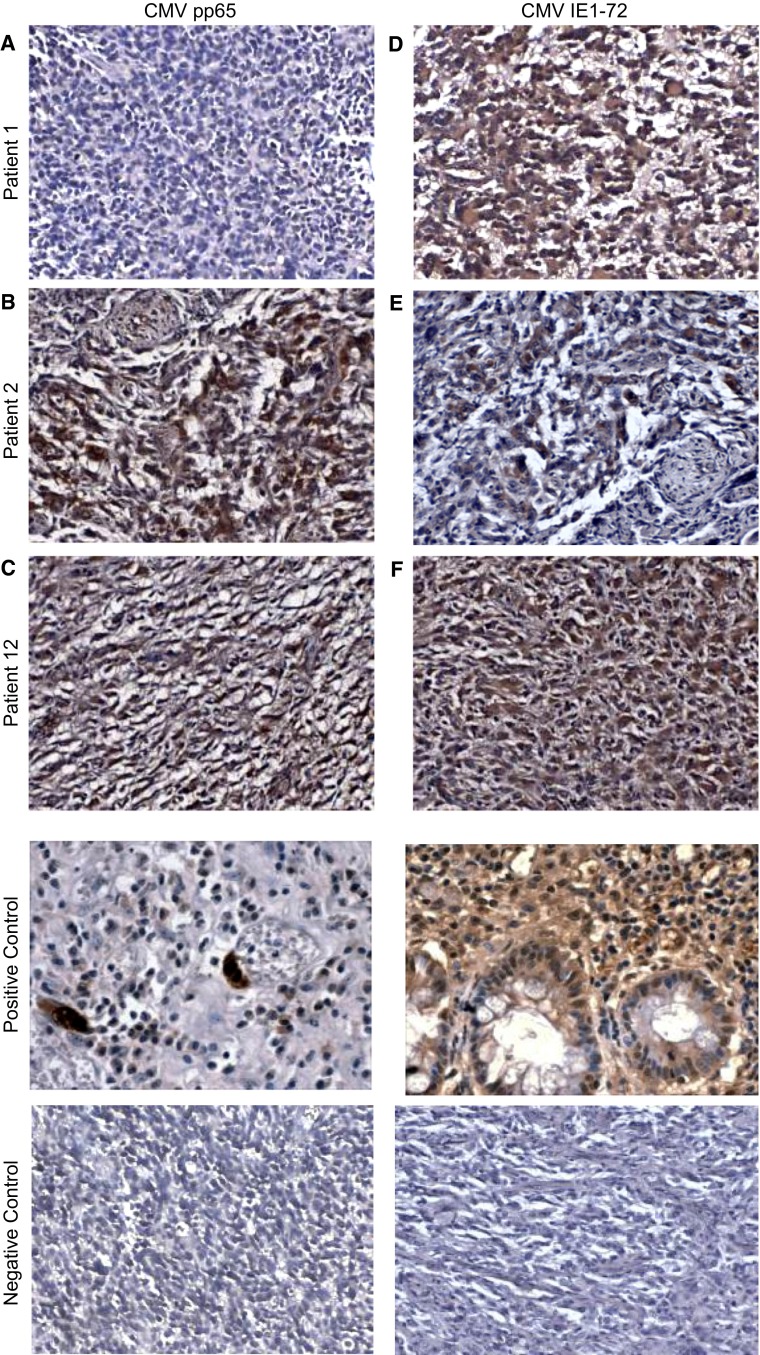
IHC for CMV pp65 and CMV IE1-72. Results from three representative patients are shown for CMV pp65 and CMV IE1-72For CMV pp65. (**a**) Patient 1 stained negative (**b**) Patient 2 stained Grade: 2 and Intensity: 2+, and (**c**) Patient 12 stained Grade: 1 and Intensity: 2+. For CMV IE1-72 (**d**) Patient 1 stained Grade: 2 and Intensity: 2+ (**e**) Patient 2 stained Grade: 2 and Intensity: 1+, and (**f**) Patient 12 stained Grade: 2 and I: 2+. Positive control is from CMV infected lung tissue and negative control has no primary antibody added. Magnification ×200. CMV positive control magnification ×400

**Fig. 3:**
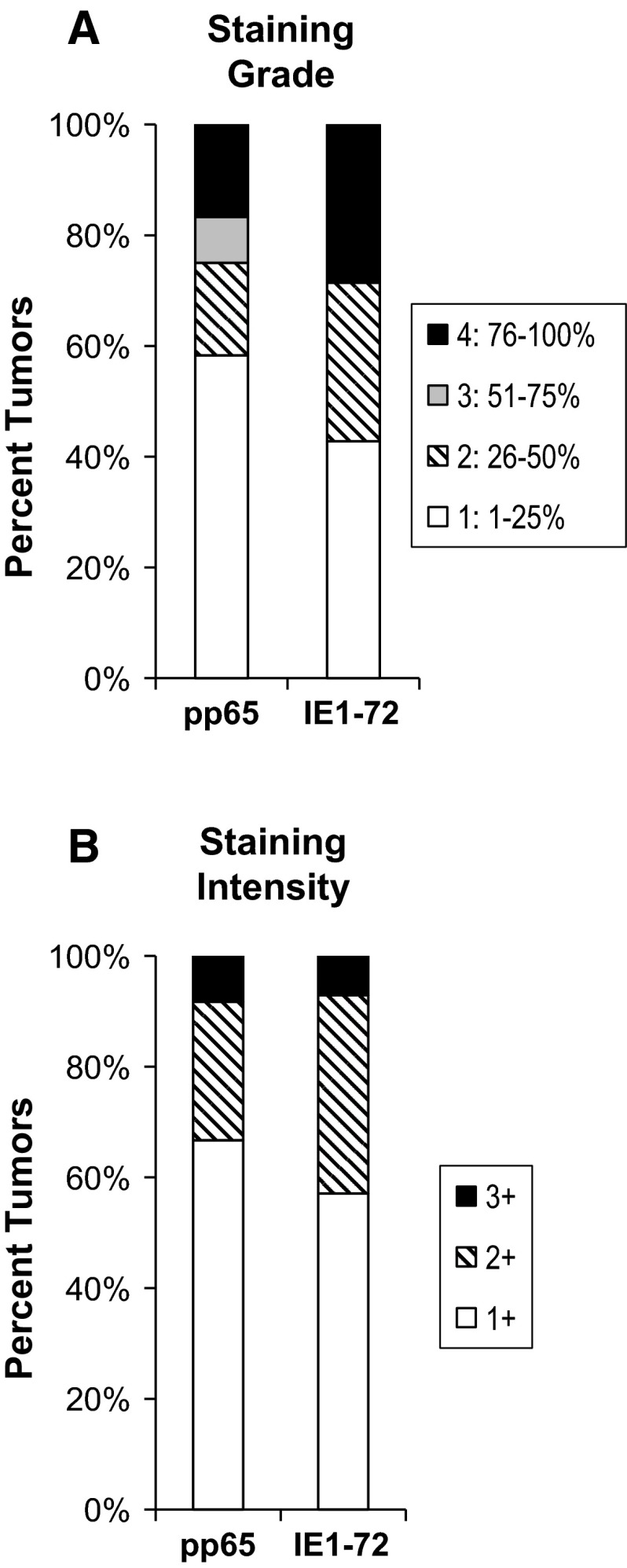
Distribution of grade and intensity of CMV pp65 and IE1-72 staining in a cohort of 25 pediatric GBM. Proportion of positive samples for each Grade (1, 2, 3 and 4) and Intensity (1+, 2+ and 3+) were analyzed for overall staining patterns. Grade and Intensity score is described in Fig. 1. Distribution of (**a**) Grade and (**b**) Intensity for samples staining positive for each CMV pp65 and CMV IE1-72 (n = 25 represents the 100 % mark)

IHC staining was performed on paraffin-embedded sections obtained from all 25 pediatric patients diagnosed with GBM (WHO grade IV). One sample was immeasurable due to extensive necrosis of tumor tissue. Sections were determined positive for CMV using antibodies specific for the CMV-encoded late protein (pp65) and CMV-encoded early protein (IE1-72) and given the grade and intensity ratings described. Photomicrographs are shown (Fig. 2) from three representative patients for CMV pp65 (Fig. 2a, b, c) and CMV IE1-72 (Fig. 2d, e, f). We found pp65 reactivity in 12 of 24 evaluable tumors and IE1-72 reactivity in 14 of 24 evaluable samples. Overall, positivity was observed for either CMV antigen in 16 of 24 patients. This data indicates CMV pp65 was observed in 50 % of our cohort, CMV IE1-72 in 58.3 %, and either CMV pp65 or CMV IE1-72 in 66.7 %. Grade and intensity of positive staining for each CMV pp65 and CMV IE1-72 were further examined to determine the staining pattern of each tested antigen (Fig. 3). Approximately 60 % of samples positive for CMV pp65 were observed as grade 1, 15 % observed as grade 2, 5 % (1 patient) observed as grade 3, and 15 % observed as grade 4. For CMV IE1-72, approximately 40 % of our samples stained as grade 1, 30 % stained as grade 2, and 30 % stained as grade 4 (Fig. 3a). Intensity distribution for both CMV pp65 and CMV IE1-72 were similar at about 60–70 % and 20–30 % staining 1+ and 2+ , respectively, and only 1 patient at 3+ (Fig. 3b).

### Expression of human CMV nucleic acid using is situ hybridization (ISH)

**Fig. 4:**
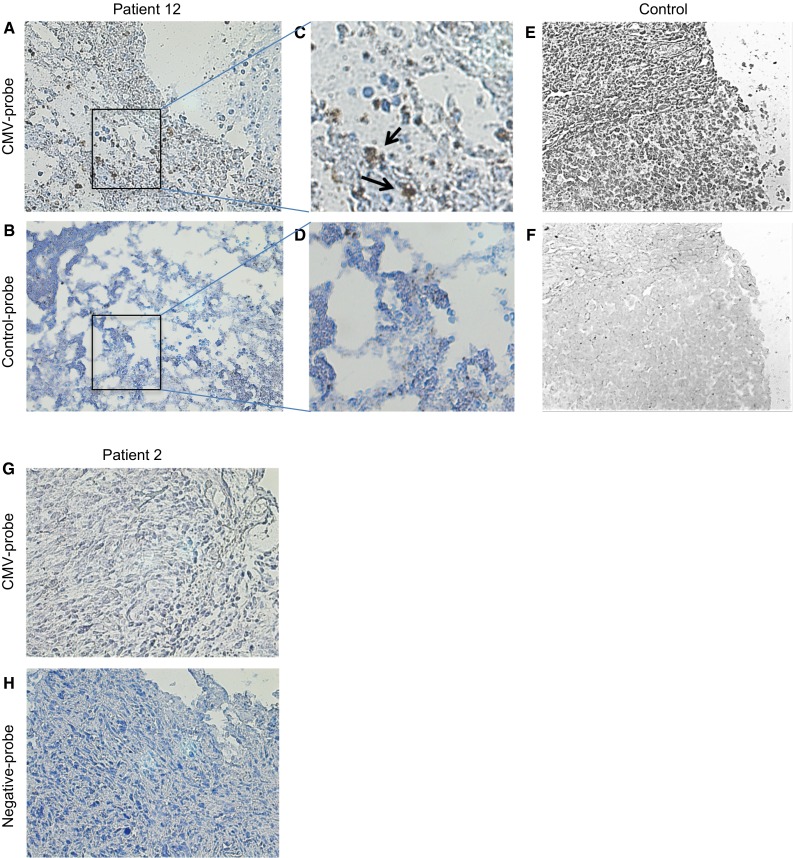
CMV genome-specific in situ hybridization (ISH). ISH was performed on 16 paraffin-embedded primary GBM samples using a CMV DNA probe. Representative results from one positive patient and one control are show. **a** Patient 12 staining positive for CMV genome using the CMV DNA probe. **b** Patient 12 staining using a negative control probe. **c**, **d** Magnification of the boxed areas in (**a**) and (**b**). **e** Staining of a positive control using the CMV DNA probe. **f** Staining of a positive control with a negative control probe. **g** Patient 2 staining negative for CMV genome using the CMV DNA probe. **h** Patient 12 staining using a negative control probe. Magnification ×100, **c**, **d** ×400

**Fig. 5:**
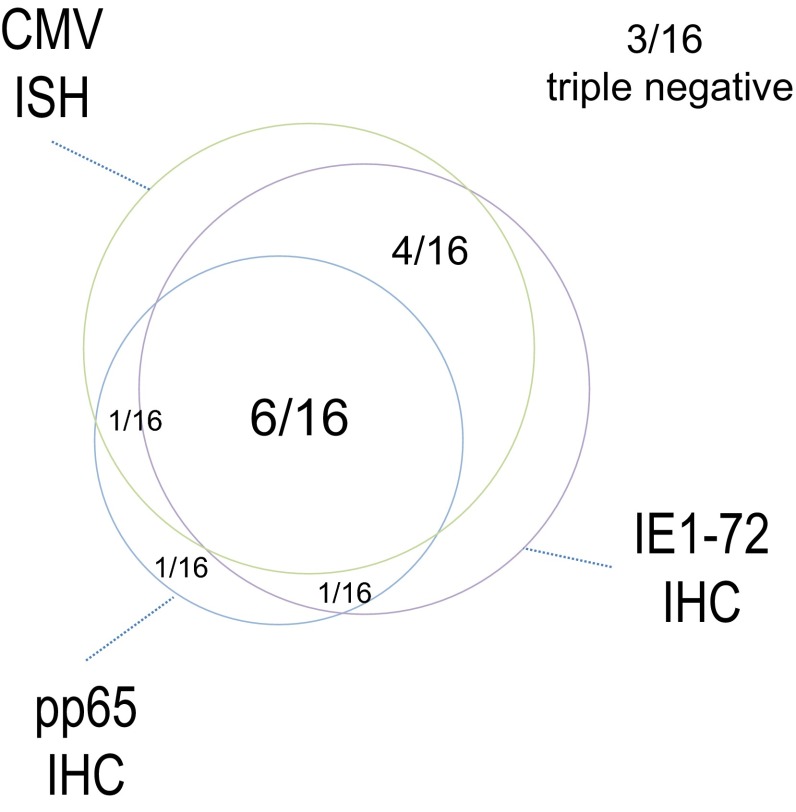
Venn diagram corroborating pp65 and IE1 detection using IHC with CMV genome specific ISH. Of a total of 16 GBMs examined using ISH, 13 were positive for CMV and 3 were negative. Eleven of 13 samples showed concomitant ISH and IE1-72 (n = 10) positivity or concomitant ISH and pp65 positivity (n = 7). Six out of 13 samples were triple ISH, IE1-72 and pp65 positive

To confirm the presence of CMV in GBMs, we performed ISH analysis using a human CMV DNA probe cocktail. Sixteen samples of the cohort of 25 were available for testing. Thirteen out of 16 GBMs were positive for the CMV genome (Fig. 4), confirming that CMV is detectable in the majority of primary GBM samples. The staining pattern was strictly nuclear for positive samples and was uniformly expressed in the majority of nuclei examined. In 11 out of 13 positive samples concomitant IE1-72 (n = 10) or pp65 (n = 7) were detected. Six out of 13 samples were triple positive for CMV nucleic acid using ISH, IE1-72 and pp65 using IHC and 3 out of 13 were triple negative for all (Fig. 5). These results confirm results for our group and those from others demonstrating a high degree of correlation between CMV nucleic acid detection and the presence of the CMV immunodominant proteins pp65 and IE1-72.

## Discussion

In this cohort of 25 pediatric serially diagnosed WHO IV GBMs, we show that either CMV antigens pp65 or IE1-72 are present in approximately 66.7 % of pediatric GBM samples. The majority of samples stained positive for either CMV antigens, pp65 or IE1-72, showing a cytoplasmic pattern in 25–50 % of cells within the sample at a moderate intensity, while a few samples showed nuclear staining and higher grade/intensity. In a sub-cohort of 16 of these pediatric GBM patients, CMV genome was detectable in 80 % of samples with a high concordance with CMV protein detection.

Using similar methodology to stain adult GBM for CMV, we previously reported approximately 45 % positivity for pp65 and 91 % positivity for IE1-72 in a cohort of adult GBM [9]. These results are consistent with previous groups reporting 50-70 % pp65 positivity in adult GBM [9] indicating a fair similarity of pp65 expression between adult and pediatric GBM. The majority of previous reports indicate a higher prevalence of IE1-72 at 90–100 % [9–12] while Lucas et al. found much lower expression at only 16 % [14]. This variation is possibly due to a difference in detection methodology and/or interpretation. Interestingly, Scheurer et al. observed 21 of 21 adult GBM samples staining positive for CMV, and saw both nuclear and cytoplasmic staining. This group also reported that approximately 79 % of cells within a sample staining positive, indicated by grade 4 in our scheme, which is much higher than our observation of only 10–20 % of samples that fell within grade 4. Our staining method was comparable to what other groups have shown for pp65 and optimized to eliminate background staining of IE1-72. Also, consistent with our observations, CMV staining in these adult cohorts was detectable primarily in the cytoplasm of GBM cells [9]. These data suggest the prevalence of CMV antigens on the majority of GBMs and indicate it could represent a potential target for novel immunotherapeutics or antivirals.

While CMV antigens offer utility as a marker whereby immune-based therapies can target GBM cells, it is possible that the ubiquitous CMV proteins identified in GBM contribute to the malignant phenotype of GBM. Therefore, targeting these molecules could potentially have a direct therapeutic benefit through disrupting disease pathways. Indeed, CMV cellular immunity (to CMV pp65), was demonstrated in a research subject after vaccination with dendritic cells that are pulsed with an autologous tumor lysate [21].

The role of CMV in the pathogenesis and propagation of GBM is the subject of ongoing research. Specifically, CMV is increasingly implicated in the pathogenesis of GBM and is being pursued as a target for cellular therapies [9, 21]. It is well known to be tropic for glial cells and is a cause of devastating encephalitis and cerebral dysgenesis in human fetuses and newborns. Interestingly, latent CMV can be reactivated in astrocytes and astrocyte-derived tumors by inflammatory stimuli [18]. Cobbs and others have shown that CMV gene products can corrupt multiple cellular pathways in GBM including mutagenesis, apoptosis avoidance, angiogenesis, and microscopic invasion [11, 13].

Other latent viruses, including EBV and human herpes virus 6 (HHV6), could behave similar to CMV when expressed on tumor cells and use natural immune system evasion mechanisms to allow tumor cells to avoid being targeted by the immune system. These would be interesting entities to investigate as potential targets in patients whose tumors do not express CMV.

Some limitations of this study include a relatively small cohort, although the largest currently reported in pediatric GBM. Further analysis of pediatric GBM patients would be warranted to furnish a better understanding of the prevalence of these antigens. IHC staining of CMV IE1-72 and pp65 is variable, however we and others have found complete concordance with ISH, as previously reported [9–12]. Indeed, when we performed ISH for CMV genome, using a cocktail of probes spanning the whole CMV genome, we found positive nuclear staining in 81 % of a sub-cohort of 16 samples. These correlated well with being CMV protein positive. Background staining has been a consistent problem with IHC for IE1-72, but our protocol was further optimized to minimize this affect with post-development incubations. Also, paraffin embedded sections that are older or not kept at ideal temperatures lose reactivity for CMV over time, potentially resulting in a lower observed level of CMV expression when compared to frozen sections or those stained immediately after resection.

Early efforts targeting both CMV-derived antigens in patients with GBM have met some success. Interestingly, retrospective non-randomized data in humans and animal models have demonstrated improved median overall survival times in hosts with GBM who receive valganciclovir [22]. Two ongoing clinical trials are exploring active immunization strategies targeting CMV-derived epitopes in patients with GBM (clinicaltrials.gov identifiers: NCT00639639, NCT00004041). Adoptive cellular therapy approaches have also been generated to target other CMV epitopes. While the roles and effector mechanisms of CMV in adult GBM are becoming better understood and targeted by cellular therapies, these advances must also be explored and translated for children, based on a sound understanding of the unique pathogenesis of GBM in these patients. Historically, treatments in children are extrapolated from adult pathophysiology or treatment data, which is often a poor correlate for disease processes and treatment responses in children. Particularly given the different molecular and genetic characteristics of pediatric GBM compared to adult GBM, a better understanding of the prevalence and function of these tumor-driving antigens in pediatric GBM is crucial to improve the poor outcomes and low overall survival rates for these children.

In this study we have shown that CMV proteins and nucleic acids are expressed on the majority of pediatric GBM samples at moderate levels. These findings, paired with the association of CMV expression with poor prognosis and overall survival, indicate the need to further investigate how these antigens are promoting tumor growth and preventing cell death. Also, the expression of these antigens in a majority of tumor tissues should be considered for immunotherapeutic targets in cases of pediatric GBM.
